# Health-Resorts in the West of England and South Wales. Bath

**Published:** 1892-12

**Authors:** A. B. Brabazon

**Affiliations:** Senior Physician, Royal Mineral Water Hospital, Bath; Medical Officer of Health


### Health-Resorts in the West of England and South Wales.

### VII.

### BATH.

BY

A. B. Brabazon, M.D.,

*Senior Physician, Royal Mineral Water Hospital, Bath; Medical Officer of Health.*

Bath, with a latitude of 51 deg. 23 min., and a longitude of 2 deg. 22 min., is 105 miles distant from London.

To the traveller as he journeys along the Great Western Iron Road to Bristol, Plymouth, or to places on the rugged Cornish coast, Bath presents a spectacle unique of its kind. Leaving behind him Chippenham, Box, with its famous tunnel, Bathford and Bathampton on the right, he speedily approaches the city. The railway, from Bathampton onward, runs along a very beautiful valley, nearly parallel with the river Avon. On his right may be seen the city, with its series of terraces of white stone houses rising one above the other, almost reaching to the breezy heights of Lansdown, culminating at Beckford's Tower, at an altitude of over 750 feet above sea level, and presenting an appearance which reminds one more of some northern Italian town than of any ordinary English town—ancient or modern.

On the left of the train speeding westwards from the village of Bathampton, may be seen Hampton and Claverton Downs, Sham Castle, Combe Down, Prior Park —famous as the former country residence of Ralph Allen, now a flourishing Roman Catholic College—Beechen Cliff, and the rapidly increasing district including Lyncombe and Widcombe, extending beyond the New Wells Road and Oldfield Park almost to Twerton.

I have travelled through many lands, and visited many health-resorts, but in none have I found so many advantages, and so few disadvantages, as in the "Queen of the West," whether regarded as a place for residence, or as a temporary resort for invalids.

The climate of Bath, generally speaking, is mild, and as to equability, neither better nor worse than that of other English health-resorts; but owing to the varieties of aspect and position of houses, a larger choice than usual can be made of residences suitable to most forms of disease and to most constitutions.

*Sanitary Conditions.*—The population of Bath, according to the last census, was 51,843. This may be taken as the resident or normal population of the "borough"; but there is a large accidental population, probably of 10,000 annually, composed of visitors who come either for baths or from curiosity to see a city which has outlived the fall of the Roman Empire as well as the various periods of decadence which has fallen upon her in mediæval and modern times. She now seems, however, in a fair way to assert her ancient dignity, not only as a health-resort, but as a residential city.

*Water Supply.*—The water supply of Bath is plentiful in quantity, and excellent in quality, slightly hard, but not in any way injuriously so. The source is from springs situated among the hills on the north and north-east of the city. There is always a large storage in reservoirs. There is now a constant supply of about 25 gallons per head daily.

The sewerage and house drainage of the city have always been under close observation, and all complaints are at once attended to; the old ashlar system of drains has been replaced by modern stoneware pipes. The house drainage is carried into the main sewers with proper intercepting traps to prevent sewer gas entering the houses. The large and never-failing water supply ensures the complete flushing of drains and sewers, and the latter are ventilated to prevent gas pressure. During the summer and autumn months the sewers are kept constantly flushed with disinfectants.

*Annual Mortality, from all Causes.*—The annual mortality of a health-resort such as Bath, which is not merely a summer or autumn resort, but is frequented by invalids at all seasons, must naturally be somewhat high, and seems in this respect to compare unfavourably with that of other places, but the peculiar conditions of Bath easily account for the fact. Firstly, as I have stated, because Bath is not a *season* resort; it is an "all the year round" place for invalids. Secondly, as I have shown elsewhere, the proportion of inhabitants over 60 years of age is excessive as compared with other towns; and though longevity in Bath is remarkable, it is somewhat counteracted by the number of people "born to Bath" at an advanced age. For the year 1891, 45 deaths were registered of persons between the ages of 60 and 80, and 95 of persons over 80. Of these, one was a centenarian, and very many were over 90. Excluding deaths in public institutions, the average quinquennial mortality from all causes, for the period ending 1891, may be fairly estimated at 16.0 per 1,000.

*Zymotic Mortality.*—The crucial test of sanitary conditions is the mortality from infectious or zymotic diseases. The average for the last five years, including 1891, is 0·4 per 1,000 annually.

These statistics, taken from the annual report of the Medical Officer of Health, speak for themselves, and need no further remark.

The small mortality is doubtless due to the existence of an Infectious Diseases Hospital, and compulsory notification. All notified cases in which isolation at home is impossible are at once removed and properly treated.

Recently there appeared in a well-known and widely-circulated weekly paper some rather caustic remarks on the "Plague Spots of England," among which unfortunate Bath has been specially referred to, on the particular grounds of the condition of the river Avon, into which the sewage of the city is discharged. There is, unhappily, no denying the fact of the sewage discharge, nor is there, in the mind of any man of common sense, a doubt as to the expediency, or, indeed, the sanitary necessity, of taking the sewage out of the river; but to carry out any scheme for this purpose the plan, to be effectual, must be adopted from the fountain-head to the final discharge of the river into the sea at Bristol. To take the sewage of Bath out of the river would be perfectly useless, in a sanitary point of view, so long as the sewage and other pollutions from the towns and cities higher up continue to be discharged into the river.

It is possible that the County Councils may in some way combine to prevent river pollution in their several districts, but that measure would require considerable consideration, and probably new legal enactments, before it could be carried into effect. Meantime, while fully acknowledging the dangers which may arise to public health by the fact of sewage discharged into a river, yet it must be remembered that there is an entirely independent water supply, and that the inhabitants of Bath do not drink the river water. If Bath is to be pronounced a plague-spot because sewage finds its way into the river, it is greatly to be feared there are many plague-spots in England, particularly where the polluted river water is drunk.

In any case, the fact has not so far much influenced the zymotic death-rate, which is infinitesimally small, though, of course, this fact is not to be used as an argument to leave matters alone. A special committee has been appointed and is now considering the most effectual means of removing any unpleasant conditions, and also the solution of a question involving an enormous expenditure of money and attended with many serious difficulties.

*The Baths of Bath, Ancient and Modern.*—The history of the Roman Baths has been written many times and oft in eloquent and classical language—so much so, that it is quite unnecessary to refer to the subject in a brief descriptive paper such as is now presented to the readers of this *Journal;* but nevertheless it is impossible to be insensible to the wonderful contrast between past and present which comes home to the mind of the visitor as he gazes on the Roman Baths over 2,000 years old, and sees lying scattered about in confusion masses of carved stone and brickwork which have successfully withstood the ravages of time, and are in as good and sound condition as on the day when they were cemented by some composition more binding than that of any modern invention; or examines the leaden pipes used to convey the *aquæ sulis* to the different baths, roughly moulded, no doubt, but still sound as ever, and, judging from the weight, composed of purer metal than that used at the present time. Yet a few yards farther, and he is within the precincts of a building recently constructed with the object of supplying every kind of bath in the best form that is obtainable by modern mechanical ingenuity, or known to modern balneological science. Doubtless these various forms of baths and modes of application of thermal waters have been copied in a great measure from Aix-les-Bains and other continental spas, but it is equally true that considerable improvements have been adopted in the modern baths of Bath which have made them second to none and superior to most of their foreign competitors.

A description of the different kinds of baths and the *modus operandi* suitable to the many and various forms of disease treated therewith would occupy too much space, but a brief survey of the bathing statistics may be interesting, as proving what has been the effect of adopting modern improvements, based on scientific grounds.

For a variety of interesting information, taken from the carefully kept records of this great bathing institution, I am indebted to the kindness of Mr. Johnston, the able superintendent of the baths. The following list of the various kinds of baths and appliances for utilisation of the thermal water generally adopted may prove of some interest; viz., deep and reclining baths, swimming baths, Aix (massage and douche), thermal vapour baths, needle douche, Scottish douche, pulverised water and inhalation rooms, medicated baths, sitz baths, Siegle spray, the bouillon and umbrella bath.

The thermal water flows from three different springs, having the same constituents, and yielding daily 507,600 gallons. They emerge from the ground at 118° to 120° Fahr. The temperature of the water in the baths is reduced by mixing with the mineral water from the large Roman Bath now exposed to view. The baths are vested in the Corporation, who have recently expended upon them upwards of £40,000. The statistics show that these modern baths are increasingly popular. The average number of baths taken during the three years ending March, 1892, was 98,537. In balneological language these mineral waters would be described as alkaline-chalybeate. An analysis by Mr. Charles Ekin shows that the water has the following number of grains of solid constituents in each gallon:

| | Grains per gallon. | | Grains per gallon. |
|---|---|---|---|
| Calcium | 28.1449 | Ammonia | 0.0175 |
| Magnesium | 3.6569 | Nitrogen as Nitrates | 0.0035 |
| Iron | 0.84 | Nitrites | Absent |
| Potassium | 2.154 | Arsenic | Traces |
| Sodium | 9.42 | Aluminium | Traces |
| Silica | 2.5012 | Lithium | Traces |
| Sulphuric Acid | 74.2915 | Strontium | Traces |
| Chlorine | 19.391 | | |
| Carbonic Acid combined | 6.16 | Total Solids | 165.2 |

*Royal Mineral Water Hospital.*—Any reference to the Bath mineral waters would be very incomplete without mention of the Royal Mineral Water Hospital. This noble charitable institution was opened more than 150

years ago. A new wing was added in 1861. The number of beds at present provided is 171, accommodating 101 males and 70 females. The charity was incorporated under an Act of Parliament in 1739. The hospital is available to inhabitants of England, Ireland, Scotland, and Wales. No recommendation from subscriber or governor is necessary. All that is required is that the case be one for which the mineral baths and waters would be suitable, and that the pecuniary circumstances would not admit of a residence in Bath while under such treatment. The hospital is undoubtedly the finest school in the world for the study of gout, rheumatism, and their congeners in every form and variety. During the year ending May 1st, 1892, 1293 patients were admitted and 1288 were discharged. Some were cured, and all were more or less benefited by the treatment. The medical staff consists of three physicians and three surgeons, and the resident house physician. The executive staff consists of the registrar and assistant-registrar, and matron. There is also a chaplain attached to the hospital.

*The Bather's Life and Amusements.*—All English resorts are severely handicapped in comparison with similar resorts on the Continent. Our insular position necessarily produces an uncertainty of climate even in summer. Nor can we boast of the beautiful scenery, enriched by mountain and lake, which surrounds many of the continental spas. The Bath season proper is supposed to extend from November to May—therefore during the worst part of the year; but the continental bathing season has the advantage of the most enjoyable part of the year, and invalids can largely enjoy not only the out-door beauties of Nature, but also the various artificial means of amusement which our continental neighbours know so well how to supply.

The invalids who resort to Bath may be divided into three classes. In the first are those who are fairly capable of enjoying life, with certain restrictions. In the second class are the invalids who, from various causes, are under strict medical *surveillance*, and cannot play fast and loose either with their complaint or with their medical adviser. In the third class are those who, having tried all remedies and all places in vain, or with very partial success, wind up with Bath as a *dernier ressort*. Possibly, in such cases, the professional adviser occasionally feels the application of the old saying when he advises his worn-out patient to "Go to Bath."

The city is fairly well supplied with public amusements, and these generally of the best kind to be had outside the Metropolis. The very pretty theatre is open all through the season, and is admirably managed by Mr. Hartree; and there is a constant succession of performances, varied in character, and carried out by thoroughly good companies. There are balls, concerts, and entertainments of various kinds at the Assembly Rooms during the winter season. Concerts, vocal and instrumental, are given daily at the Pump Room. The Bath and County Club is considered one of the best provincial clubs in England. For those of a literary fancy there is the Royal Literary and Scientific Institution, close to the Pump Room, in connection with which is a splendid Museum of Roman Remains and Geological Specimens; and a Library, containing about 5,000 volumes of valuable works, with a large Reading Room, in which will be found the daily papers and principal periodicals. The Athenæum is also a favourite literary resort, well supplied with papers and periodicals, and having a good Circulating Library. During the summer months the City Band performs twice a day, either at the Victoria Park, Sydney Gardens, or Literary Institute Gardens. There is a very good Golf Link and Club. There are Tennis Clubs, cricket, and boating on the Avon for about five miles up the river to Bradford or downwards from below Twerton to Keynsham. To the antiquarian, the geologist, and the botanist, Bath and its neighbourhood afford unbounded opportunities for study and research.

The climate of Bath is comparatively mild and equable, though, like that of all other English winter resorts, it is subject to considerable variation. Westerly winds are the most prevalent.

I give a summary of meteorological observations made at the Bath Royal Literary and Scientific Institution, and of the averages for twenty years ending 1890. This has been kindly supplied me by Mr. Henry Mitchell, Librarian.

*Estimated height above Sea Level. Barometer, 101 feet. Thermometer (verified at Greenwich), 71 feet. Rain Gauge (8 feet from the ground), 75 feet.*

| | | |
|---|---|---|
| BAROMETER— | | |
| Mean at 9 a.m. corrected to Temp. 32° and Sea Level | 29.973 | inches. |
| Absolute Maximum corrected | 30.436 | ,, |
| Absolute Minimum corrected | 29.325 | ,, |
| Range | 1.111 | ,, |
| MEAN CALCULATED FROM 9 A.M. OBSERVATION— | | |
| Temperature of the Air | 50.1° | |
| Dew point | 44.8° | |
| Relative Humidity (Saturation 100) | 83 | |
| SELF-REGISTERING THERMOMETERS— | | |
| Mean of Maxima | 56.7° | |
| Mean of Minima | 43.7° | |
| Mean Daily Range | 12.9° | |
| RAIN— | | |
| Rain and Snow | 31.802 | inches. |
| Number of Days on which .01 in. or more fell. | 163 | |

Bath has always been remarkable for educational advantages, and never more so than at the present time. Schools are numerous and excellent, and amongst them may be specially mentioned the Bath College, the High School for Girls, and the Grammar School.

Hotels and private lodgings are numerous and excellent, and as a rule the proprietors are well educated and experienced in the art of making invalids comfortable.

During May and June the city looks its best, and the surrounding country is perfectly lovely, particularly while the foliage presents that beautiful light green which is, alas! of too short duration, and soon deepens into that "emerald green" for which our *not yet* disunited sister country is famous.

